# Microfluidics for the rapid detection of *Staphylococcus aureus* using antibody-coated microspheres

**DOI:** 10.1080/21655979.2020.1831362

**Published:** 2020-10-19

**Authors:** Bo Song, Junsheng Wang, Zhijun Yan, Zhijian Liu, Xinxiang Pan, Yingbo Zhang, Xiaojie Zhang

**Affiliations:** a Department of Clinical Pathogen Biology, Medical Technology College, Qiqihar Medical University, Qiqihar, Heilongjiang, China; b Department of Information Science and Technology, Dalian Maritime University, Dalian, Liaoning, China; c Maritime College, Guangdong Ocean University, Zhanjiang, China; d Pathology college, Qiqihar Medical University

**Keywords:** Microfluidic chip, detection of *Staphylococcus aureus*, microspheres

## Abstract

*Staphylococcus aureus* is a common foodborne pathogenic microorganism which can cause food poisoning and it is pathogenic to both humans and animals. Therefore, rapid detection of *S. aureus* infection is of great significance. In this study, a microfluidic platform was introduced to detect *S. aureus* by fluorescence labeling method and a self-made microfluidic chip, which has immune spheres were used to study the effect of capturing *S. aureus*. Through this experiment, we found that the platform can be used for microbial culture, and *S. aureus* antibody coated on the diameter of 50 ~ 90 μm microspheres for detection. On the premise of optimizing the sample flow rate and detection time, the bacterial detection was quantitatively monitored. Results showed that our platform can detect *S. aureus* at injection rate of 5 μL·min^−1^ reacted for 4 min and the detection limit of bacteria is 1.5 × 10^1^ CFU/μL. However, the detection time of traditional method is 24 hs to 72 hs, and the operation is complex and cumbersome. These findings indicated that the microfluidic chip in this study is portable, sensitive, and accurate, laying a good foundation for further research on the application of rapid bacterial detection platform.

## 1. Introduction

*Staphylococcus aureus* is gram-positive rod-shaped bacterium that is ubiquitous present in the natural environment considered to be the most common food source bacterial infection [1–3]. Along with the widespread of antibiotic resistance in *S. aureus* has risen enormously, It has been characterized as a leading cause of complex infection disease which involves serious threats in both hospital and community settings for several years [4–6]. *S. aureus* causes humans and livestock bacterial infections, due to *S. aureus* can produce coagulase, so it can produce local suppurative infection disease involving mild skin and soft tissue infections, such as respiratory, pseudomembranous enteritis, endocarditis, and so on [7,8]. It can also cause blood stream infections, sepsis, toxic shock syndrome (TSS) and other systemic infections, which have severe outcomes and seriously endanger human health [9–12]. Under appropriate conditions, *S. aureus* is capable of producing enterotoxin which is destructive to the intestine and causes food poisoning, predominantly caused by contaminated food, such as milk, meat, eggs, leftovers, and so on [13,14]. Therefore, it is also an important microbial detection indicator in food. China released the national food safety standard limit of pathogenical bacteria in food (GB 29,921–2013), and formulated the limit standard of *S. aureus* in the national standard. The maximum safety limit value of acceptable level of *S. aureus* concentration should not exceed 100 CFU/g. Therefore, rapid detection and identification of *S. aureus* is crutial to prevent.

Currently, conventional microbial culture methods widely used for pathogen’s identification and quantification are still the gold standard. However, it has several limitations including time consuming that requires many hours to several days of incubation periods, complicated operation that require considerable amount of skills, and multiple equipment for analysis [15–18]. To solve this issue, up to now, many other rapid detection technicians especially for the detection and identification of bacteria such as polymerase chain reaction (PCR), loop-mediated isothermal amplification (LAMP), enzyme-linked immunosorbent assay (ELISA), quartz crystal microbalance (QCM), recombinase polymerase amplification (RPA), electrochemical impedance spectroscopy (EIS), and fluorescence spectroscopy that have been reported in the literature [15–19] to simplify the requirement of bacterial detection. Compared with traditional methods, although some exciting progress has been achieved [19–21], these methods still need to develop sensitive methods, increase the relatively high limit of detection (LOD), cut down the overall detection process and faster sample-to-result steps suitable for clinical diagnosis.

Along with the continuous development of science and technology, as a result, new rapid detection methods have been developed in attempts to search for methods that suitable for on-site detection of bacteria in many fields [16,18]. In recent years, microfluidic chip is a powerful tool technology with the characteristics of integration, miniaturization, automatic sampling, and high-throughput, which highlights the advantages of bacterial detection. Microfluidic technologies have been applied in many fields such as clinical blood diagnosis, immunology, and cancer biology [18,22,23]. However, microfluidics efficacy has not been fully demonstrated [19], such as the limit of optical detections based microfluidic systems detection is only around pg/mL [24], other methods can detect very low concentration pathogens but suffer from expensive detection and difficult to operate the chip-based assay [17,25].

In this article, the approaches based immune beads in the microfluidic have been constructed for utilizing rapid detection of *S. aureus*. The method is sensitive and specific, the report is as follows.

## 2. Materials and methods

### 2.1. Reagents and instrument

*S.aureus* (ATCC 25923), *Escherichia coli O157:H7* (GIM 1.707) and Enterococcus faecalis were purchased from Guangdong Provincial microbial species protection center (Guangdong, China) and cultured in LB broth medium(Guangdong huankai company) at 37°C for 24 h. negative photoresist (SU-8 2005 Microchem), polydimethylsiloxane (PDMS) and sylgard 184 were purchased from Dow Corning materials (USA), trimethylchlorosilane (ABCR company, Germany), AO (acridine orange). Anti *S. aureus* antibody and anti *S. aureus* antibody (FITC) were purchased from Abcam company (USA), 3-aminopropyl triethoxysilane (APTES), N-hydroxysuccinimide (NHS), 1 – (3-dimethylaminopropyl)-3-ethylcarbodiimide hydrochloride (EDC) and morpholine ethylsulfonic acid (MES) were purchased from sigma company (USA), microsphere (size 60 –90 μm) purchased from Sichuan Mianzhu biological company, plasma cleaning instrument (Chengdu Mingheng company), TS-2A four channel micro injection pump (LSP02-1B, Longer Precision Pump Co., Ltd.)

### 2.2. Methods

#### 2.2.1. Design and fabrication of microfluidic chip and antibody immobilization

The channel and structure of the microchip which is composed of two layers were designed by using Auto CAD [26,27]. The top layer is composed of four parts: entrance area, detection area, waste liquid hole and zigzag channel between entrance area and culture hole. The bottom layer was flat structure. Microcolumns were set around the detection area, that the maximum height of the micro column was slightly higher than the diameter of the microbeads. The channel was 50 mm long, 200 μm wide and 100 μm high. Mask were made according to the designed pattern and the channel template was fabricated by spin-coating su8-3025 photoresist, after silane treatment was carried out for 5 min, PDMS was poured into the chip, horizontal standing for 30 min, and baked in 80°C electric drying oven for 10 min. Then clean the silicon wafer for plasma cleaning, the upper and lower layers of chips were irreversibly bonded, and the chip production was completed [28,29].

Preparation of bacterial suspension: *S. aureus* was cultured overnight, and the bacterial solution was prepared with sterile phosphate buffer (PBS) at the concentration of 1.5 × 10^8^ CFU/μL, meanwhile the bacteria concentration was determined by plate colony count [30]. The bacterial liquid was diluted to 1.5 × 10^3^, 1.5 × 10^2^ and 1.5 × 10^1^ CFU/μL with PBS. *Escherichia coli* O157:H7 (GIM 1.707) and *Enterococcus faecalis*  were prepared by the same method as above. PBS after high pressure sterilized was used as negative control.

#### 2.2.2. Culture performance verification of microfluidic chip

The LB liquid medium was encapsulated in the microfluidic chip culture medium and the bacterial suspension with the concentration of 1.5 × 10^8^ CFU/mL was pumped into the wells of microfluidics. The liquid diffused freely to the culture chamber through the microchannel and gradually reached saturation. After incubation for 0, 2, 4, 6, 8, 10, 12, 14, and 16 h in a 37°C incubator, OD values were measured with the microplate reader (Thermo Fisher). The wavelength was set at 600 nm, and the blank medium was used as the negative control. Under the same experimental conditions, the experiment was repeated three times on different chips to calculate the average OD value. The growth curve was drawn according to OD value and culture time. Under the same experimental conditions, 96-well reaction plate was used to detect the same concentration and amount of bacterial liquid, and the accuracy of the two methods was compared.

#### 2.2.3. Activation of microspheres and preparation of immune microspheres

**Figure 1.**
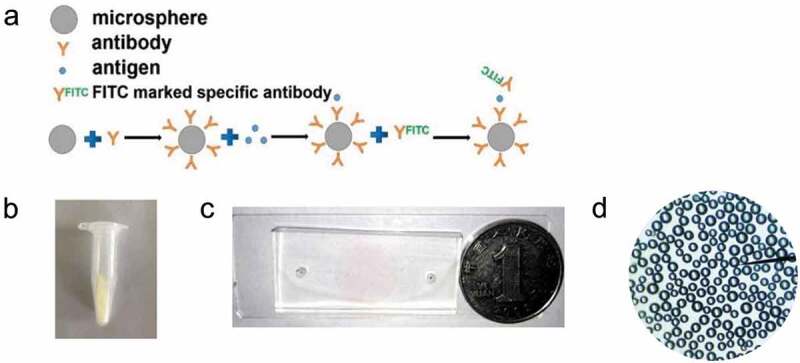
(a) The binding mode of antibody and microspheres. (b) The immune microspheres prepared were completed. (c) The diameter of sample hole and outlet of microfluidic chip is 3 mm, and the diameter of detection hole is 15 mm. The prepared chip was compared with the size of one dollar coin, (d) The microspheres under a microscope.

The surface modification and antibody immobilization of the microspheres were carried out utilizing the coupling method of sulfhydryl maleimide group [31,32]. 10 mg glass microspheres with the diameter 50 μm were accurately weighed and placed in piranha solution (H_2_SO_4_: H_2_O_2_ = 3:1) overnight, washed with distilled PBS for 5 times, dried at 70 °C for 30 min, reacted with 2% APTES acetone solution at room temperature for 30 minutes, and washed with acetone and distilled water for five times. 50 μL MES buffer, 8 μL 4 mg·mL^−1^ EDC (about 2 mmol·mL^−1^) and 12 μL 4 mg·mL^−1^ NHS (about 2 mmol·mL^−1^) were added into 10 μL antibody working solution (0.1 mg·mL^−1^). The reaction solution with 50 μg·mL^−1^ antibody concentration was obtained after 15 min reaction at room temperature. 120 μL (0.1 mol·L^−1^) PBS buffer was added to terminate the reaction. The reaction solution and microspheres were added into the centrifuge tube and reacted overnight at room temperature. The microspheres were washed three times with 0.1 mol·L^−1^ PBS [33]. The immune microspheres modified with polyclonal antibody against *S. aureus* were prepared (Figure 1). Then, the immune microspheres were injected into the microfluidic chip reaction cell by plasma pump.

#### 2.2.4. Choose the best injection rate and injection time

In this study, AO was used to stain bacteria because it can penetrate the cell wall of bacteria and combined with DNA to emit yellow green or green fluorescence. Bacteria stained with AO can verify whether the immune spheres in the enrichment chamber can effectively capture/enrich *S. aureus*. By observing the capture of bacteria by immune spheres, qualitative detection of bacteria was carried out. 1 mL AO staining solution with concentration of 10 μ g · mL^−1^ was added into 1 mL *S. aureus* solution with concentration of 0.5 MCF. The coating condition of immune spheres was judged by the fluorescence effect on the surface of microspheres under fluorescence microscope after react 1 h at room temperature.

The flow of liquid in the reaction channel was controlled by an injection pump. When the injection flow rates were 2, 5, and 10 μL/min, the intensity change of fluorescence signal in the reaction area was observed, and the optimal injection flow rate and reaction time were determined. The fluorescence signal intensity value was recorded once every 2 min until 30 min. The experimental group and the control group were injected with the chip containing immune spheres, the chip with antibody-modified spheres was used as the experimental group, while the chip filled with only 1% bovine serum albumin sealed glass beads was used as the control group. The experiment was repeated three times under the same condition.

#### 2.2.5. Detection limit and specificity of chip

1 μL of *S. aureus* suspension with the concentration of 1.5 × 10^1^ ~ 1.5 × 10^4^ CFU/μL was injected into the chip containing immune beads at the optimal flow rate. PBS was used as the negative control. After the bacterial solution reacted with the immune beads for the best reaction time, anti *S. aureus* antibody (FITC) 1 μL was added, and the fluorescence signal intensity of each detection area was analyzed by Image Pro Plus 6.0 software. The capture rate of immune beads on bacteria was calculated according to the formula: The capture rate = the number of inlet bacteria – the number of export bacteria/the number of inlet bacteria × 100%, and the detection limit of the chip was obtained. Different concentrations of *Escherichia coli* O157:H7 and *Enterococcus faecalis* were detected according to the above methods. The specificity of the microfluidic chip was determined by independent sample t test using Graph pad Prism 8 software. The experiment was repeated three times under the same condition.

#### 2.2.6. Detection of S. aureus in food samples

Two different concentrations of *S.* *aureus* suspension in drinking water were prepared. Under the optimal reaction conditions, Immunocapture and chip bioluminescence detection were carried out. Plate culture counting method was used as the concentration of control bacteria. The results of the two methods were tested by small sample t test, which confirmed that there was no statistical difference between them (P > 0.05). This indicates that the chip platform can provide accurate results for the detection of *S.* *aureus*.

## 3. Results

### 3.1. Verify the culture characteristics of microfluidic chip

**Figure 2.**
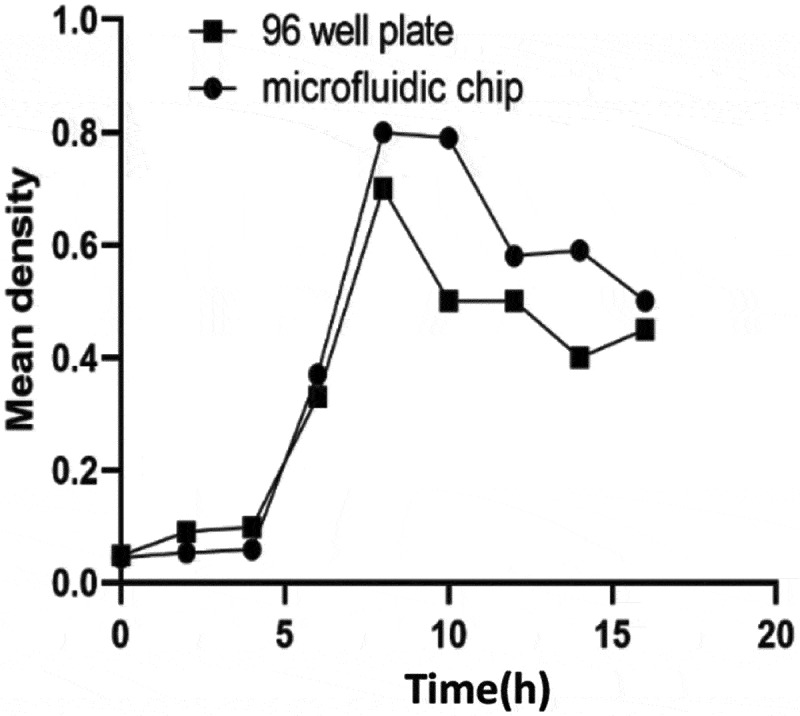
Comparative observation on the growth curve of bacteria cultured by microfluidic chip method and traditional 96 well plate method after cultured 0, 2, 4, 6, 8, 10, 12, 14, and 16 h

The growth curve of two methods of bacteria culture on microfluidic chip or 96 well plate was compared by measuring the growth curve of bacteria in 16 h. We can see that the growth curves measured by the two methods are basically the same (Figure 2). The results show that the microfluidic platform designed in this experiment is suitable for bacterial growth.

### 3.2. Determine the best injection flow rate and time

**Figure 3.**
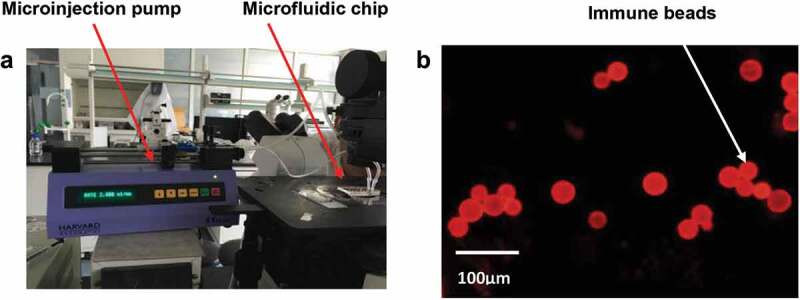
(a) When the injection flow rate was 5 μL · min^−1^, the bacteria on the surface of microspheres in the experimental group were clearly visible. (b) According to the fluorescence intensity observed by the fluorescence microscope, the time of completely capture was 4 min after reaction

When the flow rate is 2 μL·min^−1^, the fluorescence intensity of the reaction zone reaches the maximum at 10 min; when the flow rate is 5 μL·min^−1^, the fluorescence signal intensity of the reaction zone reaches the maximum at 4 min; when the flow rate is 10 μL·min^−1^, the fluorescence signal intensity of the reaction zone is within 2 min. The experimental results show that the bacteria can be completely captured at different flow rates. When the flow rate was 5 μL·min^−1^, the fluorescence signal was high and there was a long stable period. Finally, 5 μL·min^−1^ and 4 min were selected as the optimal injection rate and reaction time (Figure 3).

### 3.3. Detection limit

*S. aureus* suspension with concentration ratio of 1:1, 1:10, 1:10^2^, and 1:10^3^ was added to the experimental group, and the initial concentration of the bacterial suspension was about 1.5 × 10^4^ CFU/μL. Phosphate buffer was injected directly into the chip without antibody coating, and the measured average optical density was used as the control group. When the concentration of *S. aureus* solution is 1.5 × 10^4^ CFU/μL, the chip has a strong fluorescence intensity, which indicates that the capture rate of bacteria is high. With the continuous decrease of bacterial concentration, the fluorescence intensity decreases, indicating that the capture rate of the microshperes also decreases. When the concentration is 1.5 × 10^1^ CFU/μL, the fluorescence signal cannot be detected in the channel. According to the capture rate and *fluorescence* intensity, the detection limit of *S. aureus* was 1.5 × 10^1^ CFU/μL.

**Figure 4.**
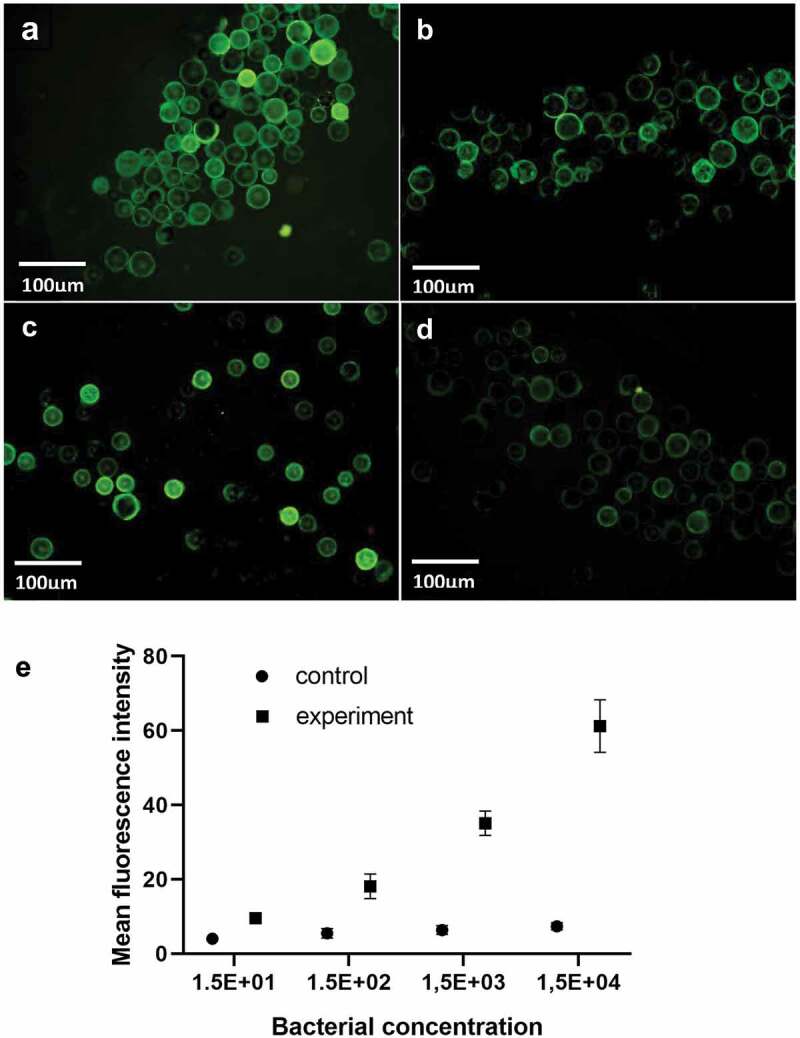
(a–d) were the *S. aureus* suspension with concentration of 1.5 × 10^1^, 1.5 × 10^2^, 1.5 × 10^3^, 1.5 × 10^4^ CFU/μl detected by fluorescence microscope (magnified by 10 × 40). (e) The standard curve of average fluorescence intensity of *S. aureus* with different concentrations.L

The photos of different concentrations of *S. aureus* suspension under fluorescence microscope are shown in Figure 4(a–d). ImageJ software was used to analyze the average optical density of the observation results. The same concentration of bacterial liquid was detected three times. At the same time, the plate count was used. The standard curve was drawn with the average optical density value as the ordinate and the number of bacterial liquid was taken as the abscissa. Figure 4(e).

### 3.4. Specificity verification

**Figure 5.**
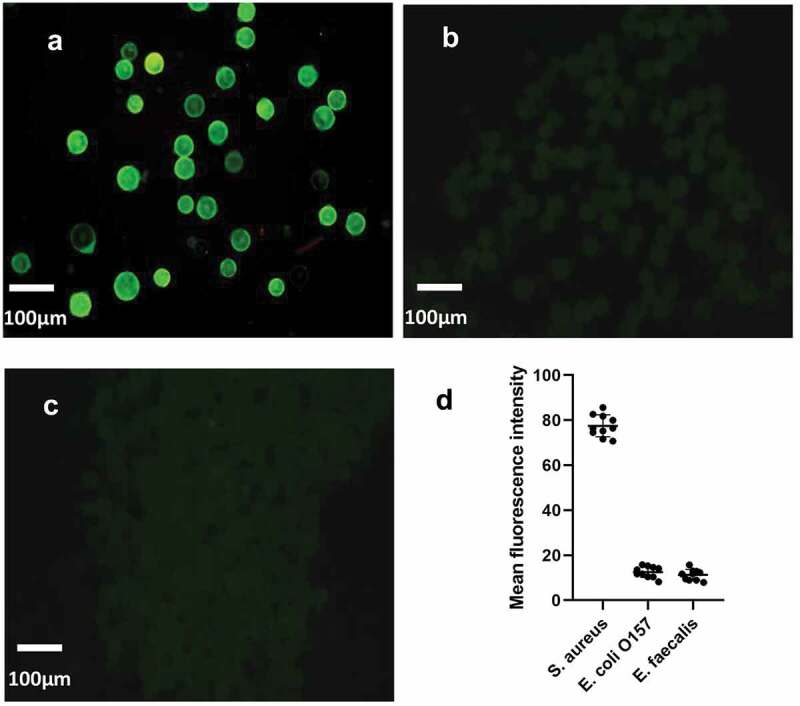
The specificity verification of microfluidic chip. The fluorescence intensity of the control group was compared with that of the experimental group. (a) was fluorescent-staining results of *S. aureus*, (b) was fluorescent-staining results of *Escherichia coli* O157: H7, (c) was fluorescent-staining results of *Enterococcus faecalis*. (d) The average fluorescence density of the three kinds of bacteria

Under the same culture and detection conditions, three different bacteria (*S. aureus, Escherichia* O157:H7 and *Enterococcus faecalis*) were detected by chip, and their fluorescence intensity was detected qualitatively, so as to verify the specificity of microfluidic chip. Through three pictures, it can be clearly seen that only the sample solution containing *S. aureus* can detect obvious fluorescence, while the *Escherichia* O157: H7(P < 0.0001, t = 37.67) and *Enterococcus faecalis* (P < 0.0001, t = 38.57) are obviously different. It indicates that the detection platform has good specificity for *S. aureus*. (Figure 5)

## 4. Discussion

In this study, we report the microfluidic platform includes sampling hole, microchannel, detection hole and waste liquid hole. At present, the culture method is often used to detect bacteria, which can identify bacteria by the color change produced by the biochemical reaction of bacteria [17,18]. However, the traditional method has the disadvantages of cumbersome operation, high cost of detection equipment, and high requirements for operators. Furthermore, there are a few reports on the use of microfluidic technology that the aptamers against bacteria was modified on microfluidic channels to detect bacteria [15]. Different in this research, the microfluidic platform is utilized with immune spheres for antigen–antibody reaction, which increases the solid surface area of antibody-modified chip and improves the capture rate [34,35]. The number of the immune spheres has a significant impact on the detection results that the number of the immune spheres is too much, it may lead to the multi-layer arrangement of immune spheres, thus the detection results will not accurate. Therefore, the shape designed of the chip and whether the immune spheres can be arranged in a single layer are very important to the detection results. In order to prevent the loss of microspheres, a micro column was set up in the detection hole, and the width of adjacent micro columns was slightly smaller than the diameter of microspheres.

In order to verify whether the microfluidic platform is suitable for bacterial culture, the growth curve of microfluidic platform in comparison with traditional 96 well plate method was observed. Our results show that the current microfluidic designed for *S. aureus* has much improved detection performance when compared with the traditional detection methods. The bacteria stained with AO were used for determining the optimal injection flow rate and he results showed that the bacteria could be completely captured at different flow rates. The fluorescence signal was high and stable when the injection flow rate was 5 μL·min^−1^, which was conducive to improve the accuracy of bacterial detection on chip. When the injection flow rate was 5 μL·min^−1^, the fluorescence signal intensity of the reaction zone reached the maximum at 4 min.

As demonstrated in this paper, the chip has the advantages of simple structure, low cost, and trace sample volume. We further demonstrate that a microfluidic chip platform, which has significant advantages in detection speed for rapid identification of pathogens is constructed. The detection time we have investigated in this study is less than 10 min, and the total reaction time is about 1 h, while the traditional method needs at least 12–48 h. The efficiency of the chip was improved by exploring the time and sensitivity of pathogen capture. The experimental foundation is established for the further development of high sensitivity and rapid detection system. The manufacturing of the whole chip is compatible with the mature micro processing technology, therefore, it has the benefit of mass produced at low cost, which is of great significance for the rapid diagnosis of such diseases.

## 5. Conclusion

Through the experiment in this study, it is founded that 1. The results show that the microfluidic platform designed in this experiment is suitable for bacterial growth. 2. 5 μL·min^−1^ and 4 min were the optimal injection rate and reaction time in this experiment while traditional culture methods need 18 to 24 hours. 3. The microfluidic chip detection limit of *S. aureus* was 1.5 × 10^1^ CFU/μL while traditional culture methods was 0.5MCF. 4. The chip designed in this experiment is semi-automatic operation, which requires low requirements for experimental operators, while the traditional method is cumbersome to operate, and the experimental personnel need to be trained to operate. 5. The chip used in this experiment is small and portable, while the traditional method needs large experimental equipment.

## Research highlights

1. This paper developed a method for the rapid detection of S. aureus.

2. The testing time can be controlled within one and a half hours.

3. The cost price of testing is about RMB 10.

4. The detection limit of this method is 1.5 × 10^1^ CFU/μL.
